# Functional Association between Regulatory RNAs and the Annexins

**DOI:** 10.3390/ijms19020591

**Published:** 2018-02-16

**Authors:** Katia Monastyrskaya

**Affiliations:** Urology Research Laboratory, Department for BioMedical Research, University of Bern, 3008 Bern, Switzerland; katia.monastyrskaia@dbmr.unibe.ch; Tel.: +41-31-632-87-76

**Keywords:** annexin, miRNA, signalling, exosome, gene expression

## Abstract

Cells respond to pathophysiological states by activation of stress-induced signalling. Regulatory non-coding microRNAs (miRNAs) often form stable feed-forward loops which ensure prolongation of the signal, contributing to sustained activation. Members of the annexin protein family act as sensors for Ca^2+^, pH, and lipid second messengers, and regulate various signalling pathways. Recently, annexins were reported to participate in feedback loops, suppressing miRNA synthesis and attenuating stress-induced dysregulation of gene expression. They can directly or indirectly associate with RNAs, and are transferred between the cells in exosomes and shed microvesicles. The ability of annexins to recruit other proteins and miRNAs into exosomes implicates them in control of cell–cell interactions, affecting the adaptive responses and remodelling processes during disease. The studies summarized in this Review point to an emerging role of annexins in influencing the synthesis, localisation, and transfer of regulatory RNAs.

## 1. Introduction

Cells respond to various physiological and pathological stimuli by activating signalling pathways, ultimately leading to changes in gene expression which constitute the adaptive responses. MicroRNAs (miRNAs) are a group of molecules, which regulate gene expression by post-transcriptional mechanisms. More than 1000 miRNAs are encoded by the human genome [1], and their expression profiles are altered in many pathologies, indicating that in addition to the developmental function, they might participate in tissue responses to physiologic and pathophysiologic stress [2]. Synthesis of miRNAs and their intercellular transfer via extracellular vesicles are also influenced by stress, and the details of these processes are not fully understood. Annexins, a family of Ca^2+^- and lipid-binding proteins, have diverse functions, but often act together as intracellular sensors of the environmental changes, allowing cells to react and adapt [3]. Recent studies, summarised in this Review, point to an emerging role of the annexin protein family, which might affect the cell signalling, by influencing miRNAs and other non-coding or coding RNAs on many levels, of their synthesis, localisation, and transport.

## 2. Annexin Expression Changes by Intracellular Stress

Cells have intricate ways of preserving the homeostasis at the times of stress, and adapt to pathophysiological states by sustained activation of intracellular signalling. Chronic stress results in profound changes in Ca^2+^ and pH, production of lipid second messengers, and lipid clustering, leading to alterations of membrane curvature. The annexins simultaneously interact with specific plasma membrane lipids and various signalling proteins [3]. The annexins are expressed in most phyla and species, and comprise a family of structurally related, Ca^2+^-sensitive proteins that bind to negatively charged phospholipids and other lipids and lipid microdomains. All annexins possess four (eight for annexin A6) conserved amino acid sequence stretches called annexin repeats, which harbour Ca^2+^ and membrane binding sites, and N-termini of variable length, contributing to specific functions of the annexin family members. Human annexins (A1–A11 and A13) participate in diverse cellular processes, such as Ca^2+^ signalling, vesicle trafficking, cell division and growth, and apoptosis regulation. Several annexins present within any one cell form a broad-range [Ca^2+^]_i_ sensing system [4]; in addition, annexin A6 and A2-S100A10 act as Ca^2+^-independent pH sensors [5]. The overlapping functions and redundant expression of annexins ensure their role as important regulators of signalling, contributing to cellular adaptation to stress signals.

Annexins act as sensors of various types of environmental changes, and often respond by alteration of their intracellular localisation or expression levels. In plant cells, annexins regulate multiple membrane trafficking events, as well as responses to extracellular signals [6]. Plant annexins mediate osmotic stress: in *Arabidopsis thaliana*, salt stress induces Ca^2+^-dependent translocation of annexins 1 and 4 from the cytosol to the membrane, and protein turnover [7]. Annexin 1 can sense the environmental pH changes, and responds by changes in ion channel conductance, hydrophobicity, secondary structure of the protein, and formation of oligomers [8]. Similarly, when the mammalian kidney epithelial cells are subjected to osmotic stress due to high sodium and chloride reabsorption, they respond by upregulation of annexins A1, A2, and A5 [9]. Annexin expression levels are adjusted to the developmental and functional state of the cell. Myoblasts undergoing differentiation after mechanical stimulation respond by elevation of synthesis of several proteins, including annexin A3 [10]. In the normal bladder urothelium, the levels of annexin A1 increase with differentiation, whereas a significant induction of annexin A6 expression is observed during urothelial de-differentiation, which might have a bearing on the regulation of proliferative responses in normal and diseased urothelium [11]. When cellular functions deteriorate, frequent changes in the expression levels of annexins have been observed [12]. Notably, significant alterations in the expression pattern of annexins are observed in many human cancers [13]. Annexins A1, A2, A4, and A6 are downregulated in prostate cancer [14], and annexin A7 is a candidate tumour suppressor gene, inhibiting prostate cell migration [15]. Similarly, annexin A3 is reduced in high-grade prostatic intraepithelial neoplasia [16]. In the bladder, annexin A6 was found upregulated in invasive bladder tumours [17]. Comparative proteomics and immunohistochemistry demonstrated that annexin A1 was upregulated in high-grade urinary bladder urothelial carcinoma, and its overexpression significantly predicted disease-specific and metastasis-free survival [18]. By contrast, it is downregulated in patients with bladder pain syndrome, which might affect cell survival and lead to the compromised urothelial function [11]. Annexin A1 is important for cell survival following mechanical or toxic damage [19], and its downregulation in urothelial cells reduced their ability to resist the exposure to a pore-forming bacterial toxin. Annexin A2 expression is increased in many cancers, including leukaemias, clear cell renal cell carcinoma, breast, cervical, colorectal, endometrial, gastric cancer, glioblastoma, hepatocellular carcinoma [20], but specifically decreased in prostate cancer [21], which might have a bearing on the regulation of cell motility in different forms of the disease. Annexin A6 acts either as a tumour suppressor or promoter, depending on the type of cancer and the degree of malignancy [22]. In several types of cancer, AnxA6 acts via Ras, [23] Ras/MAPK and/or FAK/PI3K signalling pathways, by mainly mediating PKCα, p120GAP, Bcr-Abl, and YY1 [22].

## 3. MicroRNAs—Important Stress-Induced Regulators of Gene Expression

miRNAs are quickly gaining recognition for their role in many biological processes and disease states [24,25]. Mounting evidence suggests that miRNAs are particularly important for regulating gene expression in response to physiologic and pathologic stress [26]. These 22 nucleotide-long endogenous non-coding single-stranded RNAs bind to the 3′ untranslated regions (3′ UTRs) [24,27] or, occasionally, the 5′ UTRs [28,29] or coding regions [30,31,32] of their mRNA targets, and regulate their expression. The imperfect complementarity with target mRNAs allows miRNAs to bind to multiple targets, albeit with different potency [33]. The miRNA genes are located in introns of the protein-coding or noncoding RNAs, and transcribed by RNA polymerase II as long primary precursor RNAs (pri-miRNA). Pri-miRNAs are processed in the nucleus by the Drosha–DGCR8 complex into ~70-nucleotide RNA molecules [34]. These precursor hairpin miRNAs (pre-miRNAs) are exported to the cytoplasm by Exportin-5 [35], and cleaved by the Dicer–TRBP complex into a ~20-bp miRNA/miRNA* duplex. The guide strand of the miRNA/miRNA* duplex is usually incorporated into a miRNA-induced silencing complex (miRISC), and the passenger strand or (miRNA*) is released and degraded [36]. Mature miRNAs interact with the Argonaute (Ago) proteins within the RISC complex, and direct them to the target mRNAs by binding to sites of imperfect complementarity at the 3′ UTR. The target selection is determined by the interaction of a “seed sequence” (nucleotides 2–8) at the 5′ end of a miRNA with mRNAs, which are subsequently degraded or translationally repressed [24]. While each individual miRNA only slightly regulates the expression levels of its target, usually causing less than a 2-fold change, the additive effect of multiple miRNAs, regulating the same transcript, might result in strong phenotypic alterations [26].

miRNAs are believed to play a significant role in the fine-tuning of cellular responses to stress, by mediating or modulating the stress signal, or forming parts of negative or positive feedback loops, controlling the homeostasis or intensifying the signalling. Not surprisingly, numerous miRNA profiling studies show that cancer invariably causes dysregulation of miRNA expression compared to non-tumour tissue [37]. Similarly, miRNA expression profiling in inflammatory disorders and pain points to the activation of common signalling pathways [38].

## 4. Feedback and Feed-Forward Loops between miRNAs and Annexins

Dysregulation of annexin gene expression reflects the profound disease-induced changes of cell homeostasis, and occurs concomitantly with the alteration of miRNA synthesis, spawning a number of studies, which address the role of miRNAs in the regulation of annexin expression and the impact of annexins on miRNA synthesis and function.

Annexin A1 mediates apoptosis and supresses cell proliferation [39]. In oesophageal cancers, a downregulation of annexin A1 mRNA and protein levels takes place in parallel with an upregulation of miRNA miR-196a. miR-196a showed a significant inverse correlation with annexin A1 mRNA levels in 12 cancer cell lines of oesophageal, breast, and endometrial origin, identifying miR-196a as the candidate miRNA targeting annexin A1 [40]. Binding of miR-196a to the 3′ UTR of annexin A1 was experimentally validated in a luciferase assay. By targeting and supressing annexin A1, miR-196a promoted cell proliferation, anchorage-independent growth and suppressed apoptosis, suggesting its oncogenic potential [40]. These findings were later confirmed in head and neck squamous cell carcinoma, where annexin A1 was also directly modulated by miR-196a. Annexin A1 knockdown in these cells exhibited similar phenotypic effects as miR-196a overexpression, suggesting that the oncogenic role of miR-196a may manifest itself through suppression of annexin A1 [41].

**Figure 1:**
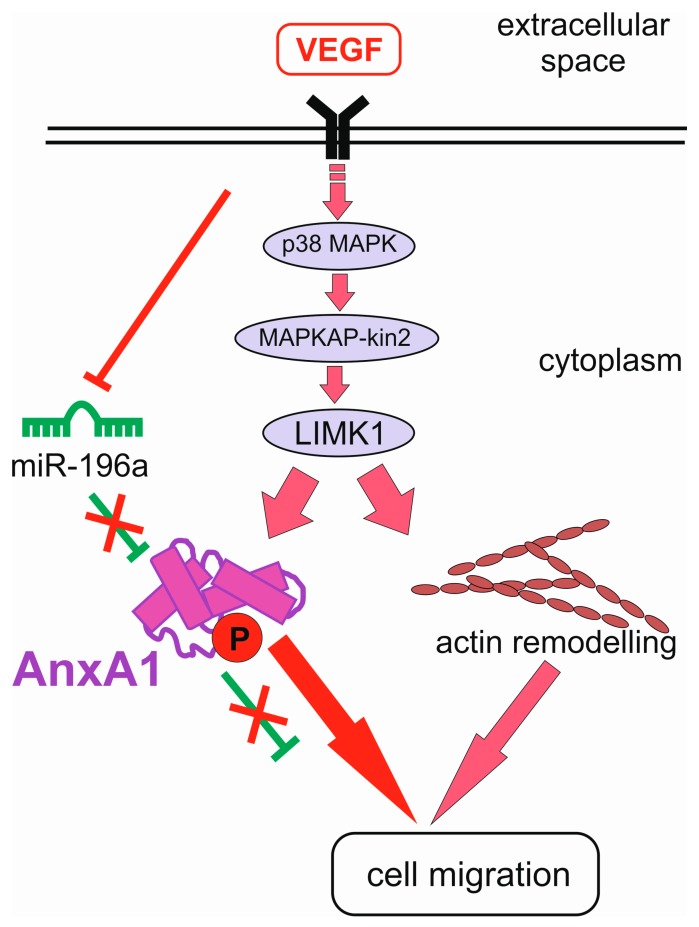
Vascular endothelial growth factor (VEGF) downregulates miR-196a to increase annexin A1. The negative feedback loop of miR-196a to annexin A1 (green T-bar) is attenuated by VEGF signalling (pink arrows), which induces a blockade of miR-196a expression (red T-bar), leading to an increase of annexin A1 and promoting endothelial cell migration.

These results indicate the existence of a miRNA-based mechanism of annexin gene downregulation in cancer, which might have consequences for the development of the disease. However, it also has a bearing on other processes, and represents a general phenomenon. The functional pairing of annexin A1 and miR-196a was demonstrated, in the context of endothelial cell migration, to be induced in response to vascular endothelial growth factor (VEGF). This essential step of angiogenesis depends, in part, on the activation of the p38/MAPKAP kinase-2/LIMK1/annexin-A1 signalling axis. When miR-196a binds to the 3′-UTR region of annexin A1 mRNA to repress its expression, decreased cell migration is observed in wound closure assays, and the inhibitory effect of miR-196a is rescued by overexpressing annexin A1 [42]. Interestingly, VEGF reduces the expression of miR-196a, which results in an increased level of annexin A1. VEGF-induced decrease of miR-196a expression may explain the angiogenic switch by maintaining the expression of annexin A1 to levels required to enable p38-annexin A1-dependent endothelial cell migration and angiogenesis in response to VEGF [42]. Additionally, VEGF-induced phosphorylation of annexin A1 downstream of the p38 pathway increases endothelial cell migration. Thus, during angiogenesis, VEGF and its effector annexin A1 form a positive feedback loop, which effectively switches off its negative regulator miR-196a (Figure 1).

Similar to the other genes, expression of miRNAs is controlled by transcription factors which represent the downstream effectors of stress-activated signalling. Within the cytoplasm, annexins associate with a wide variety of proteins, including some important hubs of stress-induced pathways. Annexin A6 interacts with the endocytic machinery and the actin cytoskeleton to inhibit epidermal growth factor receptor and Ras signalling [43], and the multiple scaffold functions may enable annexin A6 to modulate migratory cell behaviour in health and disease [44]. Annexin A1 interacts with the EGF receptor and regulates its trafficking, degradation, and signalling [45], whereas annexin A6 promotes PKCα-mediated EGFR inactivation through increased membrane targeting of PKCα and EGFR/PKCα complex formation [46]. Recently, annexin A1 was implicated in the constitutive activation of NFκB in breast cancer to promote metastasis [47]. It binds Inhibitor of Nuclear Factor Kappa B Kinase Subunit Gamma (NEMO) and Receptor Interacting Serine/Threonine Kinase 1 (RIP1) to constitutively activate the IKK complex and NFκB signalling: in annexin A1 overexpressing cells, NFκB was recruited to the CXCR4 promoter without external stimulation.

**Figure 2:**
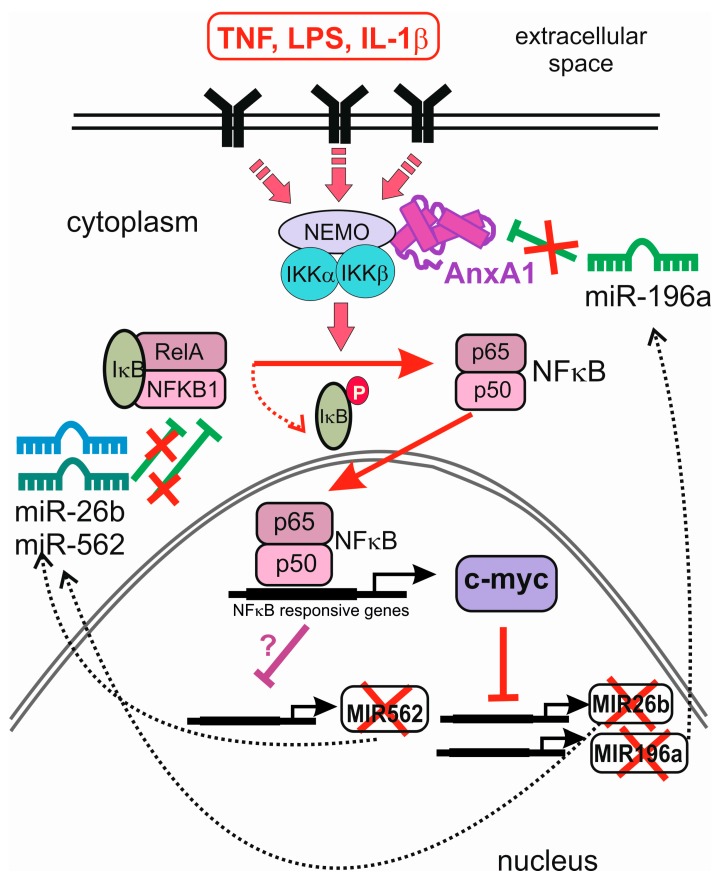
Reciprocal influence of annexin A1 and miRNAs in the regulation of NFκB signalling. Annexin A1 interacts with Inhibitor of Nuclear Factor Kappa B Kinase Subunit Gamma (NEMO) and enhances NFκB signalling (pink arrows), promoting phosphorylation and subsequent degradation of NFκB inhibitor (IκB, red dotted line) and activating transcription of NFκB-regulated c-myc. c-Myc (red T-bar) and other NFκB-responsive gene products (purple T-bar) inhibit transcription of MIR562, MIR26b, and MIR196a genes, and reduce the levels of mature miRNAs in the cytoplasm (dotted arrows). This relieves the suppressive effect of miR-26b and miR-562 on their targets, RelA and NFκB1, and the downregulation of annexin A1 by miR-196a (green T-bars, crossed). Thus, annexin A1 inhibits miRNAs miR-196a, miR-562, and miR-26b in a negative feedback loop, through NFκB and c-myc.

NFκB is involved in transcription of many miRNA genes, and it seems logical to assume that annexin A1 might influence miRNA synthesis by modulating the activity of this transcription factor. In line with its role as a NFκB-mediated gene expression modulator, a number of miRNAs, including miR-26b* (miR-26b-3p) and miR-562, were downregulated in cells overexpressing annexin A1 [48]. Interestingly, both of these miRNAs reduce NFκB activity, by targeting RelA and NFκB1 (p105), and additionally, miR-562 inhibited cell migration in a wound closure assay. The authors suggest that annexin A1-regulated miR-26b* and miR-562 may play a role in wound healing and tumour-induced endothelial cell tube formation by targeting NFκB expression. They propose that in low annexin A1 tumours, NFκB activity is reduced via a negative feedback loop involving miR-26b* and miR-562, whereas the high annexin A1 tumours display constitutively active NFκB [48] (Figure 2).

Further evidence that annexin A1 impacts oncogenic processes by altering miRNA synthesis was obtained by overexpressing this protein in breast cancer cells. As a consequence, the levels of inhibitory miR-196a were reduced, and miR biogenesis was suppressed upstream of mature and precursor miR-196a at the level of pri-mir-196a transcription [49]. This occurred indirectly through the stimulation of c-myc downstream of enhanced NFκB activity in breast cancer cells overexpressing annexin A1. In a negative feedback loop, miR-196a directly inhibits annexin A1 and increases breast cancer cell proliferation in vitro [49]. c-Myc, activated downstream of NFκB signalling, is by itself a known inhibitor of miRNA synthesis [50]. miR-26b is among the miRNAs downregulated by c-myc [50], providing a mechanistic explanation of the inhibitory role of annexin A1 in the studies mentioned above [48]. These results point to an interplay between the miRNAs, transcription factors inducing or repressing their synthesis, and annexins which indirectly affect the transcription factors’ activity. Thus, annexins might be regulating their own synthesis in feedback and feed-forward loops involving miRNAs (Figure 2).

Several other miRNAs regulating expression of different annexins have been experimentally validated: annexin A2 was shown to be targeted by miR-206 during the phenotypic modulation of pulmonary artery smooth muscle cells in hepatopulmonary syndrome [51]. Decidualization, a process associated with cell migration during embryo implantation, is accompanied by a downregulation of miR-181b-5p expression, concomitant with an upregulation of its target’s tissue inhibitors of metalloproteinase 3 (TIMP-3) and annexin A2, which are highly relevant for increased cell migration. Annexin A2 was also shown to be directly targeted by miR-1 in glioblastoma cells [52]. miR-155 is an important immunomodulatory miRNA implicated in the induction of breast, lung, liver, and prostate cancer, where it promoted cell proliferation, as well as targeted and significantly downregulated annexin A7 [53]. These studies, though not investigating the reciprocal relationships between miRNAs and the annexins which they target, nevertheless provide evidence for the functional connection between the two classes of molecules, important for cell signalling.

## 5. RNA Binding by Annexins: Implications for Localisation and Function

**Figure 3:**
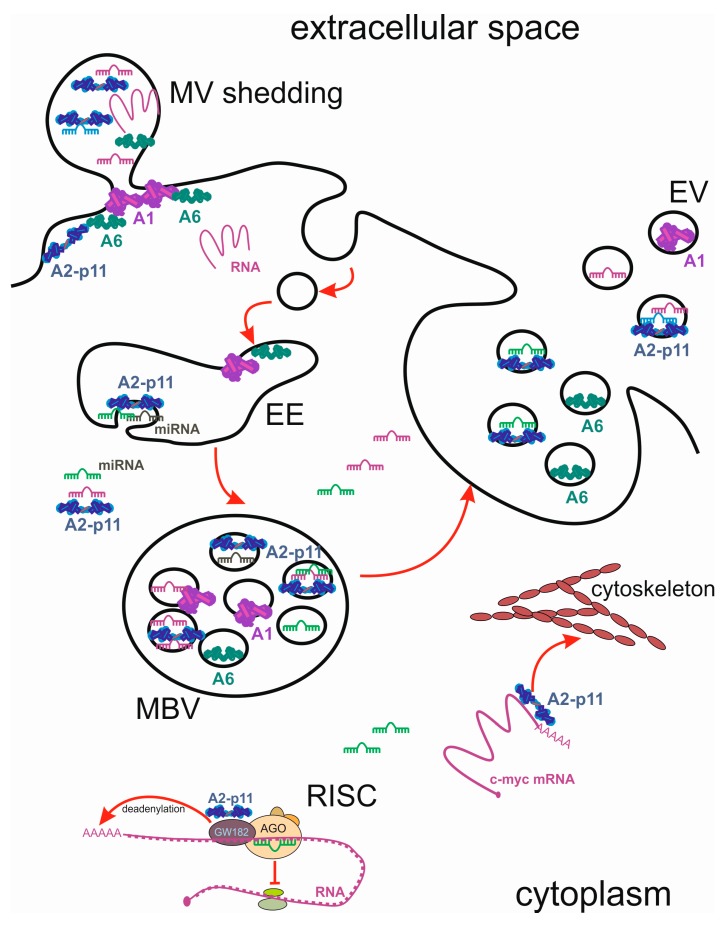
Annexins and regulatory RNAs in cellular compartments. Annexins associate with exosomes and shed microvesicles, and regulate RNA localisation and function. EE, early endosomes; MVB, multivesicular body; EV, extracellular vesicles; MV, microvesicle, depicted during shedding from the plasma membrane. Cellular processes (vesicle trafficking, cytoskeleton binding, deadenylation) are shown with red arrows.

Although annexins have traditionally been viewed as a Ca^2+^- and lipid-binding protein family, there is ample evidence of their role in RNA binding. An early study by Hirata showed that the annexin A1-S100 protein heterotetramer is a DNA and RNA binding protein capable of interacting with either purine RNAs or pyrimidine ssDNAs in a Ca^2+^- and phospholipid-dependent manner [54]. In addition to binding, annexin A1 might have a helicase activity, as its heterotetramer regulates unwinding and annealing of DNA. Unwinding occurs in a Mg^2+^- and adenosine triphosphase (ATP)-dependent manner, and annealing requires Ca^2+^ and phospholipids [55]. Annexin A2 is associated with ribonucleoprotein particles (RNPs) resembling signal recognition particles and co-immunoprecipitates with RNA fragments of apparent low sequence complexity [56]. It also binds mRNAs on cytoskeleton-attached polysomes. Binding to the 3′ UTR regions of various transcripts by annexin A2 might contribute to their specific intracellular localisation and association with the cytoskeleton. This is important for targeting RNPs to the defined cellular regions and the spatial regulation of transcription. Annexin A2 was shown to Ca^2+^-dependently bind to ribonucleotide homopolymers and human c-myc RNA with high affinity [57]. Association of c-myc mRNA with annexin A2 appears to regulate its localisation and translation: overexpression of annexins A2 in LNCaP cells resulted in the increase of c-myc protein [57], whereas a perinuclear localisation element in the 3′ UTR of c-myc mRNA, which is bound by annexin A2, guided the localisation of c-myc mRNA to the perinuclear cytoplasm [58] (Figure 3).

Annexin A2 recognises sequence elements within the untranslated regions of its cognate and the c-myc mRNAs. The annexin A2-interacting region of the 3′-untranslated region can be mapped to a sequence of about 100 nucleotides containing two repeats of the consensus sequence 5′-AA(C/G)(A/U)G. This type of interaction is representative for a group of mRNAs translated on cytoskeleton-bound polysomes [59]. The annexin A2–mRNA interaction is specific, and involves helices C and D in domain IV [60]. Positive and polar residues in helices C–D in domain IV bind to cis-acting elements in the 3′ UTRs of their cognate, c-myc, collagen prolyl 4-hydroxylase-α(I), and *N*-methyl-d-aspartate R1 mRNAs [61], thus contributing to post-transcriptional regulation of the expression of specific genes [62]. In the full-length protein, the mRNA-binding site is masked, and gains exposure through a Ca^2+^-induced conformational change.

In addition of confining mRNAs to specific subcellular locations, RNA binding by annexin A2 can have consequences for mRNA translation and affect the outcome of protein synthesis. The binding of annexin A2 to a pseudoknot structure present in infectious bronchitis viral RNA results in reduced efficiency of minus 1 (−1) ribosomal frameshifting, indicating its recruitment as a host protein during viral infection [63]. Overexpression of annexin A2 significantly reduced the frameshifting efficiency from the viral pseudoknot RNA, and knockdown of the protein strikingly increased the frameshifting efficiency [63]. Similarly, annexin A2 was implicated in the regulation of tumour suppressor protein p53, which is crucial for preventing oncogenic transformation [64]. Normally, low levels of p53 increase after stress, due to transcriptional, translational, and post-translational gene regulation. p53 mRNA has two internal ribosome entry site (IRES) elements, giving raise to full length p53 (FL-p53) and its N-terminal truncated isoform (ΔN-p53). ΔN-p53 oligomerizes with p53 protein and negatively regulates its transcriptional and growth suppressive activities. Annexin A2 and PTB associated splicing factor (PSF/SFPQ) are novel interacting proteins for p53 IRESs, especially relevant under stress conditions, when the elevation of [Ca^2+^]_i_ encourages annexin A2 binding to p53 RNA that, in turn, enhances p53 IRES function, increasing ΔN-p53 synthesis [64].

Post-translational modifications of annexin A2 are crucial for its RNA-binding activity. Serine 25 phosphorylation and ubiquitin/SUMO1 conjugation of annexin A2 induce its association with nonpolysomal mRNAs. This observation helps explain how a subpopulation of mRNAs is sequestered in a translationally inactive and transport competent form at a distinct subcellular localisation [65]. An interesting possibility for annexin A2 to influence miRNA function arises from its recent discovery as a component of P-bodies: the phosphorylated annexin A2 showed partial colocalisation with the P-body marker GW182 [65]. GW182 family proteins are essential for miRNA-mediated gene silencing in animal cells. They interact with Argonaute proteins, and are subsequently recruited to miRNA targets. GW182 proteins repress translation, and enhance turnover of target mRNAs, as well as act as scaffold proteins for the assembly of the RISC complex [66] (Figure 2). This link between annexin A2 and miRNA-mediated gene expression attenuation awaits further investigation.

Long non-coding RNAs (lncRNAs), which are described by a transcript length >200 nucleotides, comprise more than 30,000 transcripts [67]. Their functions are believed to be very diverse based on the existence of DNA, RNA, or protein-binding motifs in their sequence [68]. Growing evidence suggests that lncRNAs play regulatory roles in multiple aspects of biological processes. LncRNAs regulate apoptosis, which is particularly relevant in cancer, where they have been shown to sensitize tumour cells to apoptotic stimuli [69]. Similar to miRNAs, lncRNAs are important contributors to many immune disorders [70], further strengthening their clinical relevance. Nuclear enriched abundant transcript 1 (NEAT1), a nuclear lncRNA, has recently emerged as a key regulator involved in various physiological responses, developmental processes, and disease progression [71]. Aberrant overexpression of NEAT1 has been documented in different types of solid tumours, such as lung cancer, oesophageal cancer, colorectal cancer, and hepatocellular carcinoma, in which its high levels are associated with poor prognosis [72]. Paraspeckles are unique subnuclear structures built around NEAT1, and NEAT1 depletion with siRNAs or antisense oligonucleotides eliminated paraspeckles, whereas its overexpression promoted the generation of paraspeckles [73]. Importantly, NEAT1 lncRNAs were found to interact with paraspeckle protein components p54^nrb^/NONO, SFPQ/PSF, and PSPC1 [73]. Recently, annexin A10 was found to colocalise with the mRNA-binding proteins SFPQ and PSPC1 at paraspeckles [74]. Annexin A10 decreased paraspeckle numbers when overexpressed in HeLa cells. In addition, it relocated to dark perinucleolar caps upon transcriptional inhibition of RNA polymerase II. Downregulation of annexin A10 leads to dedifferentiation, invasion, and tumour progression, implicating this protein as a tumour suppressor [75]. In line with these data, overexpression of annexin A10 in HeLa cells increased their sensitivity to apoptosis and reduced colony formation, possibly via its membrane-independent role in paraspeckle-associated mRNA regulation or processing [74]. As most paraspeckle proteins carry a putative RNA binding domain [76], the annexin A10 core might contain a yet unidentified RNA binding site.

LncRNA-MUF (mesenchymal stem cells (MSC)-upregulated factor) is highly induced in hepatocellular carcinoma (HCC), and contributes to tumorigenesis and epithelial–mesenchymal transition (EMT). It specifically binds annexin A2 at a site between nucleotides 800 to 1600, and colocalises with annexin A2 in the cytoplasm of HCC cells [77]. The same study found annexin A2 to interact with GSK-3β, increasing β-catenin-mediated wingless-type murine mammary tumour virus integration site family (WNT) signalling and promoting EMT. Interestingly, lncRNA-MUF binds to both annexin A2 and GSK-3β via partially overlapping domains. Additionally, the cytoplasmic localisation of lncRNA-MUF is necessary for it to bind and sequester tumour-repressing miR-34a [77]. Due to its interactions with both the regulatory lncRNA and GSK-3β, annexin A2 might control EMT and promote tumorigenesis in HCC cells.

A further indication of annexin A2 involvement in HCC emerged from the studies of hepatitis C virus-induced carcinoma [78]. Annexin A2 may have an important role for the progression and treatment of viral infections due to its RNA- and protein-binding ability. It interacts with non-structural hepatitis virus proteins NS5B and NS3/NS4A, and mediates their binding to RNA, important for infectivity [78].

## 6. Intercellular Transport of miRNA and Annexins by Extracellular Vesicles (EVs)

### 6.1. Exosome Biogenesis, Release, and Uptake

The activation of intracellular signalling pathways is not the only mechanism influencing cellular levels of miRNAs and mRNAs. Circulating miRNAs have been found in body fluids, including blood serum, plasma, and urine [79,80], where they are protected from degradation by the association with lipid or protein carriers. There are numerous studies implicating them in cancer, myocardial infarction, diabetes, and atherosclerosis, and proposing their use as biomarkers for cardiovascular and haematological diseases, cancer, and CNS disorders [81,82,83,84,85]. Some of the circulating miRNAs and mRNAs are transported in high-density lipoproteins [86], the others are packaged in secreted membrane vesicles, such as exosomes and microvesicles [87], which can be actively taken up by cells, resulting in changes to gene expression [88,89,90]. Exosomes are small vesicles (30–100 nm in diameter) of endosomal origin, which are generated within multivesicular bodies (MVBs), and can be released into the extracellular medium following the fusion of MVBs with the plasma membrane. Exosomes have a unique lipid and protein composition: they are enriched in cholesterol, sphingomyelin, and ceramide, and carry a plethora of specific tetraspanins, including CD63, CD9, CD81, and CD82, as well as Rab, endosomal sorting complexes required for transport (ESCRT) proteins, Apoptosis-Linked Gene 2-Interacting Protein X (ALIX), Tsg101, and heat-shock proteins, involved in MVBs’ biogenesis and fusion [91].

Some cell types constitutively release exosomes, whereas in the others, their secretion is inducible. Ceramide is a trigger of exosomal budding into MVBs [92], and decreased ceramide production resulted in the reduced secretion of exosomes and diminished miRNA release [88,93]. Intracellular Ca^2+^ elevation is an important signal to exosomal fusion, as has been shown in cardiomyocytes stimulated with Ca^2+^-ionophore [94]. Calcium and ceramide signalling are intricately connected in the cell: intracellular Ca^2+^ overload, occurring at the conditions of cellular stress, induces ceramide production and leads to ceramide-driven segregation and internalization of membrane-associated proteins [95], which is relevant for MVB formation and exosome secretion. Hypoxia is an important feature of many pathological conditions, and HIF-1α was shown to mediate exosomal production [96]. The extracellular pH often changes under hypoxic and inflammatory conditions, and microenvironmental pH is an important factor for exosome traffic, increasing both production and vesicle uptake [97].

Docking of MVBs to the plasma membrane, resulting in fusion and exosome release, is a tightly-regulated process, involving ESCRT proteins, BIG2 [98], and Rab27A and 27B [99]. Subsequent exosomal binding to the target cells results in the release of the exosomal cargo into recipient cells, and is a limiting, but poorly understood step in molecular trafficking. In immune cells, the success of the exosomal uptake and miRNA release was shown to depend on the formation of immune synapse between activated T-cells and antigen-presenting cells, and required ceramide production by activated neutral sphingomyelinase 2 [88]. Stem cell-derived microvesicles required CD29 adhesion molecule [100]. Exosome membranes carry a selection of tetraspanins and some integrins, which are thought to be involved in vesicle fusion [101]. Some cells engulf these vesicles by endo- and phagocytosis; exosomes surf on filopodia using grabbing and pulling motions to reach endocytic hot spots at the filopodial base [102]. At least in some cases, exosome internalization is mediated by non-classical lipid-raft dependent endocytosis, rather than direct membrane fusion. This process involves ERK1/2 phosphorylation, and is negatively regulated by caveolin 1 [103].

### 6.2. miRNA Packaging and Transfer via EVs

miRNAs packaged into exosomes and shed microvesicles have been shown to influence the expression of their target genes in the recipient cells following vesicle uptake. Both mRNAs and miRNAs can be shuttled between human and mouse mast cells via exosomes, resulting in translation and appearance of mouse proteins in human cells [89]. An elegant study demonstrated that sheer stress-activated endothelial cells upregulated miRNAs miR-143/145, which were then transferred to smooth muscle cells (SMCs) via exosomes, and influenced their target gene expression, causing morphological changes in smooth muscle [90]. Similarly, microvesicles derived from human adult liver stem cells bound to hepatoma cells and inhibited their proliferation, following the transfer of selected miRNAs [100]. A number of excellent reviews summarise the current knowledge of exosome-mediated transfer of miRNA in stem cell research, cancer, and cardiovascular disease [104,105,106].

The exact molecular mechanisms of the recruitment of miRNAs into EVs are not well characterized, however, there is an indication that intracellular protein and mRNA environment might influence the miRNA packaging, and account for the differences in miRNA profiles of normal and diseased cell-derived exosomes. RNA profiling of macrophages and their exosomes showed that miRNA sorting to exosomes was modulated by cell activation-dependent changes of miRNA target levels in the producer cells. Genetically perturbing the expression of individual miRNAs or their targeted transcripts promoted bidirectional miRNA relocation from the cell cytoplasm/P bodies (sites of miRNA activity) to MVBs (sites of exosome biogenesis), and controlled miRNA sorting to exosomes [107]. Mutant Kirsten Rat Sarcoma Viral Proto-Oncogene (KRAS) colorectal cancer cells release miRNA- and protein-laden exosomes that can alter the tumour microenvironment. Mutant KRAS might regulate the composition of secreted miRNAs, leading to distinct exosomal profiles in mutant exosomes compared to wild type KRAS exosomes [108].

The observation that miRNAs form complexes with proteins has led to an assumption that miRNA-binding proteins, including annexin A2, might direct miRNAs to EVs. Indeed, annexin A2 has the ability to influence the miRNA content of EVs: silencing annexin A2 significantly decreased the amount of miRNAs in EVs without having an effect on their profiles, suggesting that it mediated the non-selective loading of miRNAs into EVs [109]. Immunoprecipitation analysis confirmed that annexin A2 could bind miRNAs in EVs in the presence of Ca^2+^. In line with the earlier observed Ca^2+^-induced increase of exosome release [94], the elevation of Ca^2+^ promoted annexin A2-mediated loading of miRNA into EVs. These results demonstrate that annexin A2 plays an important role in the sequence-independent packaging of miRNAs into EVs [109] (Figure 3).

## 7. Annexins as Stress-Induced Cargo of EVs

### 7.1. Annexins in Exosomes

Having reached the recipient cells, the contents of EVs influence their functional state, often conveying some properties of the cells of origin to the new location. EVs shed from aggressive cancer cells are capable of activating fibroblasts and altering the tumour microenvironment [110]. In human cancers, exosomes are believed to mediate the communication between stromal cells and surrounding cancer cells, leading to development of the pre-metastatic niche [104]. Annexins are powerful regulators of actin dynamics, and influence cell motility and invasiveness [111], therefore, in addition to the disease-mediated alterations of their endogenous levels, the intercellular trafficking of annexins might have consequences for the target cells. Although annexins do not have secretion signals, they are often found in the extracellular milieu: an early study showed that upon stimulation of exocytosis in chromaffin cells, a fraction of annexin A2, found inside the fused vesicles, was secreted into the culture medium [112]. The association of annexins with EVs is now believed to be an important feature of this protein family, leading to a wide range of extracellular functions. Annexins regulate many aspects of the endocytotic pathway, and associate with MVBs during their formation following receptor endocytosis, finally localising to exosomes.

A role of annexin A1 in regulation of MVBs internal vascularisation was convincingly demonstrated in EGF-stimulated cells [113]. Annexin A1 may be required for a late stage in inward vesiculation, since it operates downstream of Hrs- and ESCRT-mediated sorting, and accumulates on internal vesicles of MVB after EGF-stimulated inward vesiculation [113]. Following MVB association, endogenous annexin A1 can be released as a component of EVs: in intestinal epithelial cells, annexin A1-containing EVs activated wound repair circuits [114]. Annexin A1 has been recently described as an important component of prostasomes, membrane vesicles secreted by prostate epithelial cells, where it is found to be associated with the surface of the vesicles [115].

In-depth analysis of melanoma exosomes demonstrated their unique gene expression signatures, miRNA and proteomics profiles, compared to exosomes from normal melanocytes, including differentially expressed HAPLN1, GRP78, syntenin-1, annexin A1, and annexin A2 [116]. Importantly, normal melanocytes acquired invasion ability through molecules transported in melanoma cell-derived exosomes. Annexin A1 present in the extracellular vesicles has been shown to be necessary for their uptake by the recipient cells: endothelial microparticles, which protect target endothelial cells against apoptosis in a dose-dependent manner, carry annexin A1, while the corresponding phosphatidylserine receptor is present on endothelial target cells [117]. The uptake of microparticles by human coronary artery endothelial cells was annexin A1/phosphatidylserine receptor-dependent, and annexin A1 downregulation abrogated microparticle-mediated protection against apoptosis of endothelial target cells.

Annexin A1 exerts its anti-inflammatory and pro-resolving actions through inhibition of neutrophil trafficking and modulation of monocyte recruitment [118]. Anti-inflammatory role of annexin A1 largely depends on the extracellular protein or its N-terminal peptide interacting with the formyl peptide receptor type 2/lipoxin A4 receptor (FPR2/ALX) [119]. Treatments, increasing the release of annexin A1-containing EVs, such as all-trans retinoic acid and dexamethasone, lead to enhanced anti-inflammatory effects [120]. Released from the EVs, annexin A1 reaches the outer plasma membrane of apoptotic lymphocytes, colocalises with phosphatidylserine, and is required for efficient clearance of apoptotic cells (Figure 3).

Annexin A2-containing multivesicular endosomes fuse directly with the plasma membrane, resulting in the release of the intralumenal vesicles into the extracellular environment, which facilitates the exogenous transfer of annexin A2 from one cell to another. A significant fraction of annexin A2 is associated with endosomal membranes, and annexin A2 organised in specialised platforms contributes to nucleation, anchoring, and stabilisation of actin filaments on early endosome membranes, together with Spire1 and Arp2/3 [121]. Annexin A2 found in exosomes after gamma interferon (IFN-γ) stimulation is released extracellularly, following their fusion with plasma membrane [122]. The exosome contents can be modulated by stress or oncogenic transformation, and it has been shown that exosomes from Ras-transformed Madin-Darby Canine Kidney (MDCK) cells are reprogrammed with factors which may be capable of inducing EMT in recipient cells, including annexin A2 [123]. Similarly, annexin A2 was found to be one of the most abundant proteins in glioblastoma-derived extracellular vesicles. Annexin A2, along with its extracellular vesicle networking partners, targeted multiple pro-oncogenic signals in cells within the glioblastoma microenvironment [52].

Exosomal annexins play a role in multiple cellular processes. Exosomal annexin A2 expression is significantly higher in malignant cells than normal and premetastatic breast cancer cells. Metastatic exosomes create a favourable microenvironment for metastasis, and annexin A2 plays an important role in this process, as priming with annexin A2-depleted exosomes reduced brain and lung metastasis. Exosomal annexin A2 caused macrophage-mediated activation of the p38MAPK, NFκB, and STAT3 pathways, and increased secretion of IL6 and TNFα [124]. Importantly, increased levels of annexin A2, metalloproteinases, and diverse signalling molecules (TGF-β2, TNF1α, IL6, TSG101, Akt, ILK1, and β-catenin) were detected in exosomes secreted from prostate cancer cells under hypoxic conditions, suggesting that hypoxia-induced exosomes enhance invasiveness, stemness, and induce microenvironment changes, promoting PCA aggressiveness [125].

Annexin A6 is frequently found in exosomes, secreted by cancer cells, and, depending on the cancer types, has been ascribed varying functions, often associated with cell motility and invasiveness. Breast cancer cells secrete annexins, including annexin A6, which are predominantly cell surface-associated via the exosomal pathway. Knocking down annexin A6 in invasive BT-549 breast cancer cells was accompanied by enhanced anchorage-independent cell growth, but cell–cell cohesion, cell motility, and invasiveness were strongly inhibited [126]. The proteomic stromal signature of pancreatic ductal adenocarcinoma (PDA) shows a contribution of the annexin A6/LDL receptor-related protein 1/thrombospondin 1 (ANXA6/LRP1/TSP1) complex in tumour cell crosstalk. This complex was only observed in cancer-associated fibroblasts (CAFs), and required pathologic culture conditions that improved tumour cell survival and migration. The presence of annexin A6 on CAF-derived vesicles increased PDA aggressiveness and metastasis occurrence, highlighting this annexin as a therapeutic target and potential biomarker for PDA [127] (Figure 3).

A proteomics study of putative colorectal cancer biomarker candidates in serum extracellular vesicles identified annexin family proteins (annexin A3, A4, A5, and A11) associated with exosomes. Exosomal annexins A3, A4, and A11 were useful for detecting early-stage colorectal cancer with high sensitivity [128]. Similarly, ovarian cancer cells expressing higher levels of annexin A3 released increased numbers of exosomes, and annexin A3 was detected in exosomes released from cisplatin-resistant cells (SKOV3/Cis) by immunoblotting and immunoelectron microscopy [129].

In addition to their roles in cancer, EVs are important for accelerating blood vessel calcification in atherosclerosis. Calcification-competent EVs derived from smooth muscle cells, valvular interstitial cells, and macrophages act as the mediators of calcification in diseased heart valves and atherosclerotic plaques. The cell type-dependent enrichment of annexins A2, A5, or A6 in calcifying EVs is implicated in the regulation of EV release and calcifying potential [130]. Macrophage-derived EVs contained S100A9 and annexin A5, and facilitated hydroxyapatite nucleation, which contributed to accelerated microcalcification [131]. Vascular smooth muscle cells (VSMCs), undergoing transition from a contractile to a synthetic phenotype, contribute to atherosclerosis. Calcium stress induces dramatic changes in VSMC exosome composition and accumulation of phosphatidylserine, annexin A6, and matrix metalloproteinase-2, which converts exosomes into a nidus for calcification [132]. Elevated circulating calcium levels, found in the patients with end-stage renal disease, also increase the calcification processes by upregulating exosomal release and enriching the extracellular vesicles in annexin A6 [133].

### 7.2. Annexins in Shed Microvesicles

In addition to exosomes, cells can release other types of vesicles by direct outward budding and fission of the plasma membrane. These vesicles, termed “shed vesicles” or “microvesicles” have a variable size (ranging between 50–1000 nm in diameter) [134], and represent a distinct population of particles with a unique lipid and protein composition. Microvesicles contain high levels of cholesterol, and are characterized by the relocation of phosphatidylserine to the outer membrane leaflet [135]. Like exosomes, microvesicles are capable of mediating the transfer of proteins and RNA between neighbouring cells following their uptake by endo- or phagocytosis, and direct fusion [136]. Various stimuli can induce shedding of microvesicles, such as mechanical stress in fibroblasts [136], protein kinase C (PKC) activation [137], and the increase of intracellular Ca^2+^, for example, following activation of metabotropic and ionotropic purinergic receptors [138,139].

In fibroblasts, repeated stress and relaxation cycles induce an ectocytotic process involving transient budding of plasma membrane vesicles from the cell cortex [140]. These 200 nm diameter budding vesicles contained actin, annexin A2, annexin A6, and β1 integrin receptors, but not tubulin, vimentin, vinculin, or annexin A1. The authors deemed these vesicles analogous to the “matrix vesicles” released by chondrocytes, and postulated a role for them in extracellular matrix remodelling after wound contraction. Cells exposed to the bacterial toxin streptolysin O (SLO) sealed off “hot spots” of Ca^2+^ entry, and shed them in the form of microparticles, leading to [Ca^2+^]_i_ reduction and cell recovery. A prolonged phase of [Ca^2+^]_i_ oscillations in such cells is accompanied by a continuous shedding of microvesicles [141]. Annexins A1 and A6 were involved in the processes of membrane repair by promoting membrane segregation and fusion, resulting in the shedding of pore-containing areas [141,142]. Similar to bacterial toxins, liprotide is a complex of α-lactalbumin and oleic acid which kills transformed cells after the plasma membrane damage. The role of annexin A6 in cell repair leading to resistance of cancer cells to liprotide-induced permeabilisation was confirmed in MCF7 cells, where a knockdown of this annexin significantly increased liprotide cytotoxicity [143] (Figure 3).

An interesting study addressed the differences between microvesicle shedding and exosome release, and uptake by pulmonary microvascular endothelial cells, stimulated by high and low molecular weight hyaluronan [144]. Release of microvesicles (termed “enlargeosomes” in the study, and characterized by *AHNAK* expression) was dependent on annexin A2 expression, and their uptake increased the barrier function of endothelial cells, whereas uptake of exosomes, whose release was caveolin-enriched microdomain-dependent, had the opposite effect [144].

## 8. Conclusions

Both annexins and miRNAs constitute multicomponent systems, capable of reacting to stress events and showing long-term adaptations to disease states. miRNAs often sustain stress signalling, forming stable feed-forward loops which ensure the prolongation of the signal. Annexins, on the other hand, are part of homeostatic mechanisms, controlling the intracellular and extracellular milieu. Annexins act as sensors for Ca^2+^, pH, and lipid second messengers, and regulate various signalling pathways. They also Ca^2+^-dependently influence intracellular membrane repair, and define cell survival and proliferation under stress conditions.

Though capable of decreasing annexin expression levels, miRNAs themselves are under indirect control by the annexins, which often participate in negative feedback regulatory events, suppressing miRNA synthesis. Annexins have the ability to directly or indirectly associate with miRNAs, other non-coding RNAs, and RNA localisation sequences at the 3′ UTRs, which allows them to influence gene expression.

Intercellular communication is another area of potential reciprocal interaction between the annexins and miRNAs. Both types of molecules are found in extracellular vesicles and transferred between cells, affecting the morphology and function of the recipient cells. Intimate association of the annexins with cellular membranes, and their ability to recruit other proteins and miRNAs into exosomes implicate them as stress-mediated regulators of cell–cell interactions. Studying the reciprocal relationship between the annexins and regulatory RNAs might provide many insights into the biological functions of these two important classes of molecules.
